# Communication Challenges While Dealing With a Deaf Patient in the Emergency Department and Suggested Solutions

**DOI:** 10.7759/cureus.31091

**Published:** 2022-11-04

**Authors:** Yahia Y Akeely, Abdulhamid Q Alenezi, Norah N Albishr, Badr Ayed Almutairi, Nawaf F Alotaibi, Raghad A Almansour, Mazin A Sabi

**Affiliations:** 1 Emergency Department, Security Forces Hospital Program, Riyadh, SAU; 2 Emergency Department, Prince Abdulaziz Bin Musaed Hospital, Arar, SAU; 3 College of Medicine, Almaarefa University, Riyadh, SAU; 4 Medical Student, King Saud Bin Abdulaziz University for Health Sciences College of Medicine, Riyadh, SAU; 5 Medical Student, King Saud Bin Abdulaziz University for Health Sciences, Riyadh, SAU; 6 Faculty of Medicine, Imam Mohammed Ibn Saud University, Riyadh, SAU; 7 Emergency Department, Prince Mohammad Bin Nasser Hospital, Jizan, SAU

**Keywords:** hearing impairment, hearing difficulties, emergency medicine, deaf, communications

## Abstract

Background

In the emergency department (ED), dealing with deaf patients presents unique difficulties and obstacles. There is insufficient time to arrange for an interpreter. While the voice of the deaf patient was the focus of earlier studies, in this study, we are interested in learning about ED physicians’ difficulties and expertise. In addition, we aim to determine which approaches they suggest to address these issues.

Methodology

A cross-sectional analysis was conducted among 166 emergency physicians working in pediatric and adult departments. The data were collected from physicians working in different centers in Riyadh city from January 2022 to March 2022. The data analysis was performed using SPSS version 23 (IBM Corp., Armonk, NY, USA).

Results

In their department policy and procedures, 74.1% of participants claimed no policy or procedure for dealing with deaf patients. The majority of available communication methods were family interpreters (63.9%) and writing on paper (16.9%). Overall, 88% of respondents did not attend any training on dealing with deaf patients, despite the fact that 83.7% thought such training should be available. Furthermore, 90.4% of the participants did not know sign language. Concerning information about the Saudi Association for Hearing Impairment Services, 74.1% were unaware of such services. Concerning modern applications on smartphones, 97.6% were unaware of any existing communication app that could aid in communication with deaf patients.

Conclusions

In this study, we identified a significant deficiency in the knowledge and skills required to communicate with deaf patients. Hence, we recommend mandating education for physicians and requiring each institution to have an interpreter available 24 hours a day, either in person or via high-quality remote video.

## Introduction

Effective communication with all emergency department (ED) patients is necessary for safe management. If there are any problems or obstacles in this communication, the patient’s safety is at risk [1,2]. Deafness is one of the obstacles to communication. Some ED physicians feel anxious when interacting with hearing-impaired patients because they are unprepared. The majority of emergency physicians lack the training necessary to care for this important group of patients with unique needs [3,4].

Patients with hearing loss are divided into two groups. Deaf, with the capital letter D, are those who are deaf pre-lingual and use sign language as the primary way of communication and have a special community and culture. The other category, deaf, with a small letter d, have post-lingual hearing loss due to neurological or mechanical causes as they age. They may employ lip reading and handwriting [5].

According to the General Authority for Statistics in Saudi Arabia, Deaf persons accounted for around 1.4% (448,000) of the total population in Saudi Arabia in 2017. This number is just for Deaf persons and does not include deaf people who develop hearing problems later in life. According to King Salman Center for Disability Research’s recent statistics in 2022, there are around 80,282 Deaf people in Riyadh city [6]. According to the World Health Organization (WHO), 1.5 billion people globally live with hearing difficulties, and by 2050, the number of people with hearing impairments may reach 2.5 billion [7].

Due to the urgency of the issue, there is not enough time in the ED to request an official interpreter to attend the ED visit. Prior to such an occurrence, a plan must be established. There should be a clear policy and protocol for managing deaf patients in the ED. The majority of the time, a family member accompanies a deaf patient to the emergency room as an interpreter. This interpretation has some restrictions. Sometimes a deaf patient would not discuss a delicate matter in the presence of the family interpreter. On the other hand, the family interpreter might conceal some facts to safeguard the nearby patient who is deaf [8,9]. Therefore, it is preferable to have a trained interpreter who is familiar with medical terminology and is employed by the institution itself.

Prior studies in the literature have addressed the viewpoint of deaf patients [1,2,4,10]. The purpose of this study is to determine the physician’s perspective on communication with deaf patients. To our knowledge, no similar study has been conducted in the literature that addresses the physicians’ point of view and the related obstacles.

## Materials and methods

Study design, period, and setting

This cross-sectional analysis was approved by the Institutional Review Board (IRB) of the Security Forces Hospital Program (research number: 21-553-66). The Strengthening the Reporting of Observational Studies in Epidemiology (STROBE) recommendations were adhered to in this study. We included all physicians working in the emergency room at multiple centers in Riyadh because we wanted to get better results that show the broad range of physician experiences. A response rate of 63% was obtained when the questionnaire was administered to ED physicians. Between the months of January and March 2022, the questionnaire was sent out to and collected from the designated physicians.

Study participants

All physicians working in the 10 hospitals located in Riyadh city were eligible. Eligible candidates included any physician working for a minimum of two years in the ED. Emergency physicians specializing in both pediatric and adult care were included. Due to a lack of previous experience working with deaf patients, physicians working primarily in the administration were not permitted to participate in the study. Everyone who took part in the study gave consent after being fully informed.

Data collection

To verify the accuracy of the questionnaire, a preliminary study was conducted. It was developed and distributed to a group of emergency physicians with different levels of professional experience to solicit their feedback on the clarity and significance of each question. Subsequently, the questionnaire was redrafted and resent to the respondents. Once the final significant requirement for clarity of each item was met, the questionnaire was approved for widespread distribution.

Through the use of email, the questionnaire was sent out to all relevant medical professionals. They were emergency physicians who specialized in both children and adults. They filled out the questionnaire which was subsequently checked for completeness and to ensure there were no missing data in the responses. The response rate was 65%.

Study questionnaire

The questionnaire contained information about the physician’s demographics, such as age, working level, gender, and age, as well as the name of the hospital. Concerning the accessibility of the policy and procedure, a question was added. In addition, it included a direct question regarding the presence of difficulty in previous communication experiences, as well as the mode of communication that was utilized. The questionnaire asked about learning, courses taken, and experience with dealing and communicating with deaf patients. It also included questions about respondents’ willingness to participate in various training sessions and courses. Awareness of sign language, courses currently being offered, and services currently being provided by deaf-related societies and associations were also enquired about. This survey also asked respondents about their familiarity with any smartphone applications as well as any newly developed communication technologies.

Statistical analysis

Data analysis was performed using SPSS version 23 (IBM Corp., Armonk, NY, USA). Frequency and percentages were used to display categorical variables. The chi-square test was used to test for the presence of an association between categorical variables. The comparison was done to determine whether the physician’s age, sex, or work history would influence their willingness to learn sign language.

## Results

A total of 166 participants were included in the study. Table 1 shows the sociodemographic profile of the participants and their place of work. Overall, 80 (48.2%) participants were less than 30 years old, 64 (38.6%) were 31-40 years old, 15 (9%) were 41-50 years old, and seven (4.2%) were 51 years and older.

**Table 1:** Sociodemographic profile of the Participants and their working place (n = 166).

Demographical characteristics	n	%
Age		
Less than 30 years	80	48.20
31–40 years	64	38.60
41–50 years	15	9.00
51 years and older	7	4.20
Gender		
Male	109	65.70
Female	57	34.30
Hospital		
King Fahd Medical City (KFMC)	38	22.90
Prince Sultan Military Medical City (PSMMC)	31	18.70
King Saud Medical City (KSMC)	22	13.30
King Khalid University Hospital (KKUH)	21	12.70
Prince Mohammed bin Abdulaziz Hospital (PMAH)	17	10.20
Al Iman General Hospital (AGH)	14	8.40
National Guard Hospital (NGH)	12	7.20
Security Forces Hospital (SFH)	11	6.60

**Figure 1:**
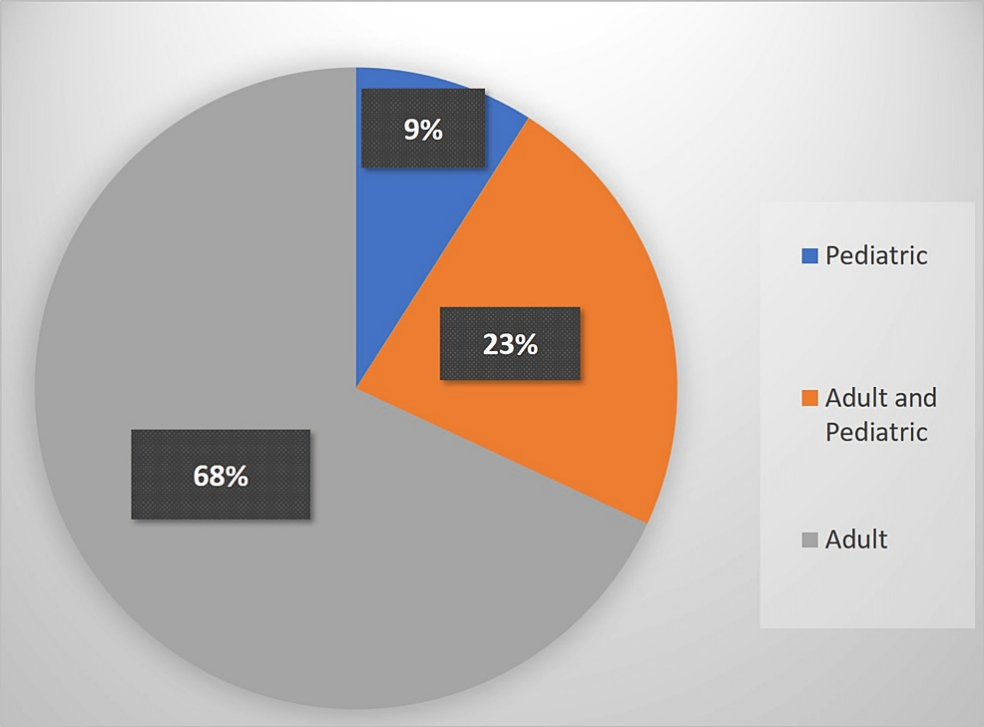
Participants’ departments.

Figure 1 displays the working department of the participants. In total, 15 (9%) participants were working in the pediatric department, 113 (68.1%) were working in the adult department, and 38 (22.9%) were working in both the adult and pediatric departments.

**Figure 2:**
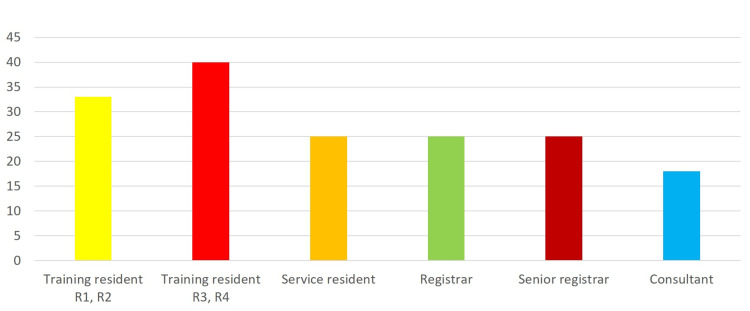
Participants’ working level.

Figure 2 presents the working level. In total, 33 (21.1%) participants were training residents R1-R2, 40 (25.3%) were training residents R3-R4, 25 (15.1%) were service residents, 25 (15.1%) were registrars, 25 (12.7%) were senior registrars, and 18 (10.8%) were consultants.

Table 2 illustrates the participants’ awareness, perception, experience, and attitude toward deafness. Only 25 (15.1%) participants reported having a policy and procedure in their hospital regarding communication with a deaf patient, and 123 (74.1%) reported facing difficulties in communicating with deaf patients. Regarding how the participants communicated with deaf patients, the most commonly reported means were having one of the family members as an interpreter for 106 (63.9%), the patient himself/herself writing on paper for 28 (16.9%), and having a patient’s friend as an interpreter for 10 (6%) participants. The other ways of communication with less occurrence with lip reading and video emote interpreter. In total, 20 (12%) participants reported that they attended teaching sessions/courses about dealing and communicating with a deaf patient, and 139 (83.7%) reported that they believe they should have a training session about communication with deaf patients. Among the participants, 16 (9.6%) reported that they know sign language, 43 (25.9%) reported that know about the Saudi Association for Hearing Impairment Services, and four (2.4%) reported they know an application they can use on smartphones to help better communicate with deaf patients.

**Table 2:** Participants’ awareness, perception, experience, and attitude toward deafness (n = 166).

Question	n	%
Do you have a policy and procedure in your hospital regarding communication with deaf patients?		
Yes	25	15.1
No	45	27.1
I’m not sure	96	57.8
Did you have communication difficulty with a deaf patient before?		
Yes	123	74.1
No	43	25.9
How did you communicate with a deaf patient?		
One of the family as an interpreter	106	63.9
A patient’s friend as an interpreter	10	6
Official hospital staff interpreter	8	4.8
Telecommunication video with an interpreter	6	3.6
Patient himself/herself writing on a paper	28	16.9
The deaf patient understands the lipreading	3	1.8
Another way	3	1.8
I know the basics of sign language	2	1.2
Did you attend any teaching sessions/courses about dealing and communicating with a deaf patient?		
Yes	20	12
No	146	88
Do you believe that we should have a training session about communication with deaf patients?		
Yes	139	83.7
No	27	16.3
Do you know the sign language of deaf people?		
Yes	16	9.6
No	150	90.4
Are you willing to know sign language if there is a free teaching course?		
Yes	140	84.3
No	26	15.7
Do you know about the Saudi Association for Hearing Impairment Services?		
Yes	43	25.90
No	123	74.10
Do you know any application you can use on your smartphone to help you better communicate with deaf patients?		
Yes	4	2.4
No	162	97.6

Table 3 shows the factors associated with the participants’ willingness to learn sign language. Age, gender, working department, and working level were all not significantly associated with the willingness to learn sign language. Most participants regardless of their levels, sex, and age were willing to learn and have more teaching sessions in sign language. The majority wanted to have better solutions and plans to deal and communicate with deaf patients.

**Table 3:** Factors associated with participants’ willingness to learn sign language. *: significant at level 0.05.

Factor	Participants’ willingness to learn sign language		P-value
	Willing to learn	Not willing to learn	
Age			0.653
Less than 30 years	65 (81.3%)	15 (18.8%)	
31–40 years	55 (85.9%)	9 (14.1%)	
41–50 years	14 (93.3%)	1 (6.7%)	
51 years and older	6 (85.7%)	1 (14.3%)	
Gender			0.167
Male	95 (87.2%)	14 (12.8%)	
Female	45 (78.9%)	12 (21.1%)	
Working department			0.966
In the pediatric emergency	13 (86.7%)	2 (13.3%)	
In the adults emergency	95 (84.1%)	18 (15.9%)	
Both	32 (84.2%)	6 (15.8%)	
Working level			0.566
Training resident R1 or R2	29 (82.9%)	6 (17.1%)	
Training resident R3 or R4	35 (83.3%)	7 (16.7%)	
Service resident	23 (92%)	2 (8%)	
Registrar	16 (76.2%)	2 (8%)	
Senior registrar	16 (76.2%)	5 (23.8%)	
Consultant	14 (77.8%)	4 (22.2%)	

## Discussion

The majority of participants in this survey reported difficulty communicating with deaf patients. Therefore, it highlights the critical need for change, as well as the implementation of new solutions and policies.

According to our findings, the predominant method of communicating with deaf patients is via a family member. As previously mentioned, family interpreters may have trouble accessing more private and sensitive information. It is always ideal to have a certified interpreter who is familiar with medical terminology which supports the findings of the study by Shuler et al. [8]. A small group of clinicians attended a course on communicating with and treating deaf people. In addition, the majority of participants were interested in taking such courses. Few participants are aware that any smartphone application can be used to interact with deaf patients. In addition, just a few of them were aware of the services supplied by Saudi Arabia’s general deaf-related organizations.

The majority of participants’ departments lacked explicit policies and procedures regarding how to interact with deaf and hard-of-hearing patients. Infrequently, lipreading, video remote interpretation, and official hospital interpretation were utilized. In this study, the deficiencies in teaching, course attendance, and sign language competency were identified.

The findings of this study support earlier research addressing unsatisfactory communication with patients who are deaf [11-13]. The strategies and actions for improved deaf patient communication based on our own and prior research are listed in Table 4.

**Table 4:** Suggested solutions for better communication with deaf patients in the emergency department.

Solutions
Determine the patient’s preferred method of communication
As much as feasible, conduct the interview in a quiet environment
Use body language at all times
Constantly maintain eye contact
Know the department’s policies and procedures, as well as the resources available in such situations
Avoid leaving the patient alone with no caregiver
If possible, utilize handwriting and lipreading, but be aware of each method’s limitations
Note the communication methods used during this visit
Review the patient’s file and any available medical reports in great detail
Perform additional physical examinations and make use of bedside ultrasound
Before proceeding with any procedure or physical examination, provide a thorough explanation
Utilize accessible smartphone applications, as specified in your department’s policies and procedures, to facilitate communication
Use images whenever possible
Use a clear, calm tone and avoid shouting
If a qualified professional interpreter is not available, use a family member or a friend instead
Ascertain that the patient understands the discussion and repeat the key points

It is important to inquire about the patient’s preferred or best accessible mode of communication [14]. In addition, assuring a quiet environment with few distractions is important. Face-to-face conversation with eye contact should be preferred. Moreover, it is important to utilize body language. In addition, repeat the facts, summarize the concerns, and ensure that they understand the discussion completely [1,3].

Furthermore, an official and trained interpreter should be available in the hospital for physical attendance or via high-fidelity video telecommunication services [15-18]. In addition, emergency physicians and medical students should receive training on how to communicate with deaf patients [19]. It should include all aspects of deaf patients, not just sign language. For instance, gaining a deeper comprehension of their culture, becoming more aware of their needs, and learning about the services offered by the government’s organizations are important [20].

We suggest that the Saudi Central Board for Accreditation of Health Institutions compel each institution to have a trained and qualified interpreter as a condition for complete certification. During a patient’s visit, the communication procedure should be recorded and documented.

It is important to talk about the deaf patient’s emotions and ideas. It is a great chance to inquire about patients’ experiences with abuse and screen them for any issues that need to be addressed and reported to healthcare professionals [19]. It is important to be aware of the delicate matters and particular considerations that the deaf population faces.

The use of sign language has evolved with time, just like any other language. Additionally, it has changed as a result of the widespread adoption of smartphones. A brand-new global sign language has emerged [21].

Video remote interpreting technology can be utilized in the ED if an on-site interpreter is unavailable or takes time to arrive. To ensure patient and physician satisfaction, the video should have a good resolution quality and a fast internet connection.

One of the strengths of the study is its multicenter design; it encompasses a number of the most important hospitals in Riyadh, which allows for a good generalization of the findings. On the other hand, one of the limitations of the study was that it did not include interviews with deaf patients themselves; however, this topic has been extensively covered in prior research, and the primary focus of this investigation is on the physicians themselves, as well as the challenges they face from their point of view.

## Conclusions

In this study, we found that a high percentage of candidates lacked the necessary knowledge and skills to communicate with and care for deaf patients. One of the three most important aspects of improving communication with deaf patients is physician education about dealing with deaf patients through well-organized lectures or courses. The second critical aspect is that each department should have clear policies and procedures in place that outline how to interact with and handle all concerns with deaf patients in the ED. The third aspect is to have a qualified interpreter available in the ED 24 hours a day, seven days a week, either physically or through well-established video-assisted communication.
